# Sex Differences in Emotions and Eating Behaviors among People Affected by Obesity

**DOI:** 10.3390/brainsci12121663

**Published:** 2022-12-03

**Authors:** Carmela Mento, Amelia Rizzo, Antonio Bruno, Maria Catena Silvestri, Clemente Cedro, Iman Komaei, Giuseppe Navarra, Maria Rosaria Anna Muscatello

**Affiliations:** 1 Department of Biomedical, Dental Sciences and Morpho-Functional Imaging, University Hospital of Messina “G. Martino”, 98125 Messina, Italy; 2 Psychiatric Unit, University Hospital of Messina “G. Martino”, 98125 Messina, Italy; 3 Department of General Surgery, University Hospital of Messina “G. Martino”, 98125 Messina, Italy

**Keywords:** obesity, emotional eating, sex differences, bariatric surgery

## Abstract

Relatively little research has examined sex differences among people affected by obesity. The aim of this study is to assess the relationship between negative emotions and eating behaviors, taking into account the role of biological sex. The final sample consists of 200 candidates for bariatric surgery, 62 males (31%) and 138 females (69%), aged from 18 to 60 years (M = 40.71; SD = 11.30). Participants were screened with the Binge Scale Questionnaire (BSQ) and individually evaluated with the Eating Disorder Inventory (EDI) and the Profile of Mood States (POMS). Correlations were calculated by splitting the sample by sex. Analyses of the relationship between negative emotions and eating behavior showed a large number of correlations in the sample of women and few correlations in men. The differences between women and men with obesity suggest the need for a different theoretical construct that explains the differentiated mechanisms of functioning and lays the foundations for specific therapeutic paths.

## 1. Introduction

### 1.1. Theorethical Standpoint on Gender Gap Differences

Male and female obesity rates have been estimated to be similar. A 10-year observational study estimated, however, that the male population undergoes significantly less bariatric surgery. The number of male patients undergoing bariatric surgery remains consistently lower than female population and has higher health risks, higher mortality, higher disease severity, and higher comorbidities [1].

Despite this, in the post-surgery comparison, males with obesity report greater psychological wellbeing, in terms of levels of depression, body satisfaction and body image compared to females with obesity [2].

The authors speculate that there are physiological and psychological differences that may explain this gender gap. However, the literature findings are sometimes conflicting. One study showed that, sometimes, men may have a gender disparity in care access, due to demographics and socioeconomic background. It has been estimated that approximately 80% of patients undergoing bariatric surgery are female [3].

Furthermore, in the population of men with obesity, the motivation to lose weight is more correlated with regaining greater effectiveness in performance and having a better chance of keeping a job [4].

According to Cohn et al. [5], there are various motivations for undergoing the intervention and these mainly relate to: concerns about one’s own health, feelings of hopelessness due to lack of control over food, feelings of inability to control eating habits, personal identity (self-blame).

Usually, females were more likely to report a history of depression and/or anxiety, and scored significantly higher than men on depression and social anxiety measurements [6].

However, both women and men affected by obesity, compared to the general population, tended to experience more feelings of anger towards the self, with concurrent higher levels of suppression [7].

Therefore, this disparity in care access is not clear from a psychological point of view, and the differences found in the literature only partially explain the cognitions and emotions related to eating behavior, which evidently lead men and women suffering from obesity to different health behaviors and levels of body satisfaction.

Indeed, a recent review has shown that body image after surgery may not change, as the patient’s outward image or the physiological parameters of the BMI calculation do not correspond to the inner experience of the person with obesity even after a dramatic weight loss [8].

Furthermore, it has not yet been clarified why after surgery some patients report psycho-social wellbeing and satisfaction and others do not. Therefore, it is crucial to study individual differences in order to understand whether the psychological mechanisms of men and women with obesity are different and deserve different psychological treatments.

### 1.2. Eating as a Way to Cope with Stressors

Several pieces of the literature report that difficulties in emotion regulation can represent a psychological vulnerability factor involved in the development of eating disorders. These difficulties in maintaining a healthy lifestyle or failing in weight control generally result in weight gain. In turn, weight and body mass index (BMI) have been found associated with negative emotions, mood alterations, and low self-esteem [9].

Some authors assumed that negative affectivity could precede and maintain maladaptive eating behavior. Some eating behaviors such as uncontrolled eating, emotional eating and restrained eating, are common among women with obesity and may affect weight loss outcomes [10].

The potential mechanism by which emotion regulation relates to obesity would be the concept of “eating as a way to cope with stressors”, i.e., negative emotions. Emotional eating is the tendency to eat in response to stress or negative emotion. Food intake is an attempt to face or avoid distressing and unwanted emotional states. Unfortunately, loss of control, overeating and weight gain, from a psychological point of view, have a negative emotional impact, arousing emotions such as guilt, shame, anger and self-disgust. Given the vulnerability in the tolerance of negative affections/emotions, a vicious cycle is established [11].

Several theories have been used to explain emotion regulation in obesity from a cognitive-behavioral perspective. The theory of emotional eating considers eating behavior as a coping strategy aimed at facing emotional distress, thus suggesting the presence of a sub-clinical form of “emotional eaters” [12].

A more recent comparison study showed that individuals with obesity reported more emotion regulation difficulties and less interoceptive awareness than normal weight control groups; participants with obesity were characterized by a lack of planning strategies and lower Emotional Awareness, as well as by a diminished ability to observe, notice and trust bodily sensations [13].

However, empirical evidence of the comparison of these dimensions among male and female population affected by obesity is lacking. Based on this theoretical background and on clinical observations, it is hypothesized that males and females affected by obesity could have different cognitive, emotional and behavioral layouts. In the light of this hypothesis, the aim of this study is to identify the correlations between negative emotions and eating behaviors by comparing the two groups based on sex.

## 2. Materials and Methods

### 2.1. Sample

The whole sample consisted of 250 individuals suffering from obesity, consecutively recruited and evaluated during the pre-surgical psychiatric assessment at the Psychiatry Unit of the University Hospital of Messina, Italy, from November 2018 to December 2019. Patients were invited to complete a booklet containing anagraphic data, a measure of eating behavior and a measure for negative emotions, described in detail in the instrument section. Data collections were associated to a clinical interview, conducted by a psychologist and a psychiatrist.

Inclusion criteria were: (a) age ≥ 18 years and <60 years; (b) absence of significant current and/or lifetime psychiatric disorders which temporarily or permanently contraindicate bariatric surgery, such as intellectual disability, mood disorders, psychosis, substance abuse disorder, anorexia nervosa, bulimia nervosa, binge eating disorder (BED); (c) agreement to research purposes with a written informed consent. All the patients provided written informed consent after a full explanation of the protocol design, which had been approved by the local committee for ethics and was conducted according to the Declaration of Helsinki. Anonymity was granted according to the Italian law for personal information treatment.

Data regarding personal data, total and subtotals of the scales administered were entered in an Excel^®^ data sheet to lead statistical analysis. Of the initial pool, 12 cases with missing values have been excluded from the analysis, 38 (F = 31; M = 7) have been excluded because they met the criteria for the diagnosis of BED, resulting in a total of 200 valid cases, consisting of 62 males (31%) and 138 females (69%) aged from 18 to 60 years old (M = 40.71; SD = 11.30).

### 2.2. Instruments and Procedure

In the present study, the Binge Scale Questionnaire (BSQ) was employed to detect the presence of BED. The BSQ is indicated to provide quantitative information on the severity of attitudes and the typical behaviors of bulimia. It was mainly used as a screening indicator for patient exclusion. Conventionally, a cut-off score >9 could indicate the presence of an eating-related problem [14].

For the psychological assessment of eating behaviors, the Eating Disorder Inventory (EDI) has been used. The EDI is composed of 64 questions, divided into eight subscales. Each question is measured on a six-point scale, with answers ranging from ‘always’ to ‘never’, rated 0–3. The score for each sub-scale is then summed. The eight EDI subscales are: (1) Drive for thinness (DT): excessive concern with dieting, preoccupation with weight, and fear of weight gain; (2) Bulimia: episodes of binge eating and purging; (3) Body dissatisfaction: not being satisfied with one’s physical appearance; (4) Ineffectiveness: Assesses feelings of inadequacy, insecurity, worthlessness and having no control over their lives; (5) Perfectionism: not being satisfied with anything less than perfect; (6) Interpersonal distrust: reluctance to create close relationships; (7) Interoceptive awareness (IA): “measures the ability of an individual to discriminate between sensations and feelings, and between the sensations of hunger and satiety”; (8) Maturity fears: the fear of facing the demands of adult life [15].

For the assessment of negative emotions, the Profile of Mood States (POMS) was used. The questionnaire, a standard validated psychological test formulated by McNair, Lorr & Droppleman [16] contains 58 words/statements that describe feelings. For each item, the subject must indicate on a Likert scale from 0 (not at all) to 4 (very much), the level of that feeling experienced in the last week [16]. The instrument consists of six subscales: (1) Tension—Anxiety, (2) Depression—Dejection; (3) Aggression—Anger, (4) Vigor—Activity, (5) Fatigue—Indolence; (6) Confusion—Bewilderment. The subject obtains a score for each subscale, which can be transformed into standard scores (points T) to be compared to the norm. Scores on average 50 ± 10 are conventionally considered normal.

## 3. Results

### 3.1. Statistical Analysis

Descriptive statistics were used to summarize demographic factors. Continuous data were expressed as mean ± standard deviation (S.D.); non-continuous data were expressed as frequency and percentages. Statistical analyses were performed with Statistical Package for the Social Sciences—SPSS 25.0 software (SPSS Inc., Chicago, IL, USA) single user license. Correlational analysis (Pearson’s correlation) was performed to evaluate the possible correlation between negative emotions and eating behavior according to gender. After Bonferroni correction, a *p*-value < 0.003 was considered statistically significant.

### 3.2. Sociodemographic and Clinical Data

With regards to their sociodemographic characteristics, the professional status of the majority of the sample was unemployed or housewife (51%), followed by 47% of employed and 2% of retired. The main educational level was 8 years (47%), followed by 13 years (38%) and >13 years (11%), only 4% of the sample reported a low educational level (5 years). Concerning marital status 63% were married, 24% single, 11% divorced and 2% widowed. Descriptive statistics showed that 67% of the sample were classified as “very severely obese” (III class), whereas 33% were “severely obese” (class II). Before performing the statistical analyses, it has been verified if age or BMI severity could have an impact on results variance using the ANOVA test. The test revealed no significant differences due to age or BMI classifications.

### 3.3. Gender Differences in Eating Behavior and Negative Emotions

Table 1 shows the Student’s *t*-test for gender-based groups means comparison. Findings show several significant differences between male and female.

The medium total score for the subscales measuring Drive for thinness, Body dissatisfaction, Ineffectiveness was higher in women, indicating a more compromised and pathological eating behavior (*p* < 0.001). Nevertheless, women also reported higher interoceptive awareness than men (*p* < 0.001).

Subsequently Student’s *t*-test has been performed in order to verify any difference in mood profile between women and men. Among the pool of negative states, only Tension/anxiety resulted significantly different between genders, being higher in women (Male M = 44.35 SD = 7.14; Female M= 50.36 SD = 11.35 *p* < 0.001). Nevertheless, this mean value falls within the normal range (40–60 T points).

In order to verify our hypothesis on possible different ways of functioning between women and men in negative emotions and eating behavior, the sample was divided in two groups according to gender, and Pearson’s bivariate correlations were performed for each group.

Table 2 shows correlations between POMS and EDI scores among women. All EDI subscales, except for Maturity Fears, were significantly correlated with POMS scales. In detail, levels of anxiety, depression, anger, fatigue and confusion were all positively correlated with eating disorder facets: the higher the intensity of negative emotions, the higher the drive for thinness, bulimic behavior, body dissatisfaction, sense of ineffectiveness, maladaptive perfectionism, interpersonal distrust, and interoceptive non-awareness. Contrarily, negative correlations were found among the vigor subscale and the eating disorder dimensions, thus indicating that higher levels of disordered eating behavior and weight concerns were associated with low energy.

Table 3 shows Person’s bivariate correlations between POMS and EDI scores in the male group. In this case, the correlations among the examined variables are more occasional. The most consistent results are: (1) Positive relationships between Bulimia and the levels of tension and depression; (2) Positive correlations between the sense of ineffectiveness and all the negative emotional spectrum, with the exception for vigor; (3) Positive relationships between low interoceptive awareness and all subscales, with the exception of vigor. Furthermore, among males, high levels of energy resulted associated with Perfectionism.

To further test sex differences, the odds ratio between each negative emotion and eating behavior stratified by sex was calculated. In the model, correlations between eating behavior and negative emotion that were statistically significant in women population were considered as events in the exposed group, while relationships between eating behavior and emotion that were not significant were considered non-events in the exposed group. Conversely, significant correlations between eating attitudes and negative emotions among men were considered as events in the control group and non-significant correlations were entered as non-events in the control group. The results of the calculation showed an Odds Ratio (Exp/Control) = 8.076 with a Confidence Interval set at 95% [3.244, 20.110]; Left-Sided Interval [3.756, +∞]; Right-Sided Interval [−∞, 17.366]; Z-score = 4.488 and *p*-value 0.000004.

## 4. Discussion

Our findings showed that women with obesity had a more compromised and pathological eating behavior than men with obesity. This is in line with the literature, which has emphasized that women are generally more dissatisfied with their body image than men [17]. In a recent study by Voges et al. (2019), the authors found that this difference could depend on the application of double standards. In their experiment, the authors found that men were more able than women to self-enhance their own body image with a beneficial effect on self-worth and body satisfaction [18]. Furthermore, Rand & Wright (2001) found a more restrictive thinness as the ideal standard body image for women and a heavier ideal body size for men. However, the quoted gender studies have been carried out on normal-weight subjects. It can be hypothesized that similar cognitive biases can also be present in subjects affected by obesity, but there is no empirical evidence supporting this hypothesis [19].

Concerning sex differences between subjects affected by obesity in mood profiles, according to previous study by Chauvet-Gelinier et al. (2019) obesity has been associated with depression and anxiety [20]. In line with previous studies, we found that levels of self-reported anxiety and depression are significantly higher in women. This is consistent with the systematic literature review conducted by De Wit et al. (2010), who included seventeen studies for a total of 204.507 participants affected by obesity. The authors found a significant positive association between depression and obesity in the general population, and this correlation was stronger in women [21].

The main result, however, concerns the relationship between negative emotions and eating behavior and how they interact in the population of women vs. men affected by obesity. In general, in women, negative emotions and the psychopathology of eating behavior are strictly associated and tend to overlap. The high number of correlations obtained from this study support the existence of a very close relationship between food and emotions, providing an empirical confirmation of the theory of emotional eating even among women affected by obesity. This result is consistent with other studies that found higher levels of emotional eating in women affected by obesity, compared with normal or underweight women [22].

Regarding the male subsample, the few correlations among the variables studied could suggest a different psychological functioning for what concerns emotions and eating behavior.

The first aspects characterizing the functioning of the male population with obesity were the relationships among Bulimic behavior (such as item 28. “I ended up bingeing every time I realized I couldn’t control myself” or item 46. “I eat moderately in front of others and gorge myself when they are gone”) and the levels of anxiety and depression. These findings could indicate an association between negative emotions and eating-related feelings, such as loss of control and shame. In other words, the loss of control would be associated with feelings of demoralization and nervousness. However, bidirectionality has not clarified whether negative emotions may have preceded or followed the loss of control in men. Psychological aspects including negative emotions (e.g., depression, anxiety, anger) that lead to maladaptive eating behaviors can contribute to and exacerbate obesity [11].

The second important correlation found in men with obesity was among the sense of ineffectiveness and the entire negative emotional spectrum, with the exception of vigor. This result might indicate a passive attitude towards undesirable emotion, and it can take the form of a belief of an external locus of control of problems. Often patients affected by obesity are bariatric surgery candidates who consider surgery and sleeve gastrectomy as a “magic bullet” for weight loss. This could depend on the underlying belief of personal ineffectiveness towards weight related problems, probably due to repeated attempts and failures in body weight control and emotional regulation [23].

The third peculiar finding in the male group was the presence of positive correlations among low interoceptive awareness and the entire negative emotional spectrum, except for vigor/energy. As seen, interoceptive awareness is defined as the sense of physiological condition of the body which can be conscious or unconscious. In this context, interoception plays a key role in enhancing self-regulation, especially in distinguishing hunger and appetite from feelings and emotional states such as anger and sadness. It is consolidated that IA is a key feature of eating disorders, and also present in overweight and subjects with obesity [24], although these associations have not always been consistent. Merwin and colleagues (2010) argued that interoceptive awareness includes both acceptance of affective experience and clarity regarding emotional responses. The authors found that problems of eating behavior are not so much linked to lack of clarity but are better explained by the non-acceptance of emotions [25].

## 5. Limitations

Our findings should be interpreted in the context of important limitations. The first limitation is the under-representation of the male sample. Second, it is possible that the strong motivation to be suitable for the surgical intervention might have prompted the subjects to belittle their psychological problems. Therefore, our findings may under-represent the psychosocial impact of bariatric surgery.

A bias on sample selection is the age range, as the psychological needs are different in the various age groups. These points deserve future explorations.

## 6. Conclusions

The general aim of this study was to find evidence of the sex differences in the relationships between negative emotions and eating behavior among subjects with obesity. There are several implications of our study.

The present findings add further empirical data to the literature on male subjects with obesity, a population often neglected, scarcely represented or maybe less willing to participate in research studies. Moreover, males and females present with different psychological and psychotherapeutic needs.

Therapists and mental health professionals have at their disposal evidence-based manuals such as CBT-OB therapy [26]. In light of the results, however, some reflections can be raised. With regards to the population of women suffering from obesity, the results suggest that they might benefit from cognitive-behavioral therapies focusing more on emotional regulation such as Marsha Linhean’s DBT, especially for “suffering tolerance” and “crisis survival” skills [27]. With regards to men with obesity, who have different characteristics and specific needs, a programme that increases interoceptive awareness, such as Mindfulness-Based Therapy, and the Mindful Eating module, might be more indicated [28]. Awareness of individual differences can also point to increasingly targeted pre- and post-surgical prevention and may decrease prejudice and care disparity [29].

## Figures and Tables

**Table 1:** Gender differences at the Eating Disorder Inventory—EDI.

	Male		Female		*t*-Test for Equality of Means	
EDI	Mean	SD	Mean	SD	T	Sig. (2-Tailed)
Drive for thinness	6.15	4.28	8.63	4.84	−3.643	0.000 *
Bulimia	1.45	2.09	2.20	2.86	−2.084	0.039
Body dissatisfaction	13.03	6.00	17.49	6.16	−4.812	0.000 *
Ineffectiveness	1.73	2.54	3.67	4.44	−3.898	0.000 *
Perfectionism	4.39	3.74	4.38	3.28	0.019	0.985
Interpersonal distrust	4.23	3.42	4.85	3.93	−1.133	0.259
Interoceptive awareness	2.26	2.93	4.28	4.54	−3.754	0.000 *
Maturity fears	7.66	5.05	7.80	4.37	−0.193	0.847

* *p* < 0.003

**Table 2:** Correlations between negative emotions (POMS) and eating behavior among females (N = 138).

	Tension	Depression	Aggression	Vigor	Fatigue	Confusion
Drive for thinness	0.429 **	0.362 **	0.357 **	−0.291 **	0.328 **	0.310 **
Bulimia	0.277 **	0.206	0.304 **	−0.117	0.267 **	0.278 **
Body dissatisfaction	0.309 **	0.275 **	0.277 **	−0.280 **	0.248 **	0.227
Ineffectiveness	0.459 **	0.613 **	0.499 **	−0.404 **	0.494 **	0.485 **
Perfectionism	0.273 **	0.280 **	0.303 **	−0.086	0.197	0.178
Interpersonal distrust	0.361 **	0.359 **	0.340 **	−0.297 **	0.294 **	0.350 **
Interoceptive awarness	0.481 **	0.471 **	0.476 **	−0.238 **	0.468 **	0.441 **
Maturity fears	0.164	0.198	0.179	−0.244	0.165	0.234

Legend: ** *p* < 0.003.

**Table 3:** Correlations between negative emotions and eating behavior among male (N = 62).

	Tension	Depression	Aggression	Vigor	Fatigue	Confusion
Drive for thinness	0.144	0.079	0.045	0.272	−0.015	−0.142
Bulimia	0.399 *	0.378 *	0.151	−0.077	0.338	0.225
Body dissatisfaction	0.181	0.221	0.162	0.000	0.209	−0.029
Ineffectiveness	0.488 *	0.657 *	0.432 *	−0.303	0.557 *	0.490 *
Perfectionism	−0.113	−0.123	−0.132	0.464 *	−0.159	−0.297
Interpersonal distrust	0.137	0.183	0.078	0.073	0.125	0.102
Interoceptive awarness	0.494 *	0.575 *	0.352 *	−0.175	0.504 *	0.418 *
Maturity fears	0.144	0.169	0.049	0.155	0.137	0.094

Legend: * *p* < 0.003.

## Data Availability

The study did not report any data.
